# Integrative network toxicology and molecular docking reveal 4-Nonylphenol’s multifaceted mechanisms in breast cancer pathogenesis

**DOI:** 10.1371/journal.pone.0331944

**Published:** 2025-09-09

**Authors:** Congli Jia, Fu Yang

**Affiliations:** 1 Department of Plastic Surgery, Plastic Surgery Hospital of Shandong Second Medical University, Weifang, Shandong, China; 2 Department of Hepatobiliary Surgery, The First Affiliated Hospital of Kunming Medical University, Kunming, Yunnan, China; Columbia University Irving Medical Center, UNITED STATES OF AMERICA

## Abstract

**Objective:**

This study employs integrated network toxicology and molecular docking to investigate the molecular basis underlying 4-nonylphenol (4-NP)-mediated enhancement of breast cancer susceptibility.

**Methods:**

We integrated data from multiple databases, including ChEMBL, STITCH, Swiss Target Prediction, GeneCards, OMIM and TTD. Core compound-disease-associated target genes were identified through Protein-Protein Interaction (PPI) network analysis. Gene Ontology (GO) and Kyoto Encyclopedia of Genes and Genomes (KEGG) enrichment analyses were subsequently employed to elucidate the potential molecular functions and biological pathways associated with these key targets. Molecular docking using AutoDock Vina was conducted to investigate the binding interactions between the core genes and 4-NP. Furthermore, the miRDB database was utilized to identify potential microRNAs (miRNAs) that may exert regulatory control over the pivotal genes.

**Results:**

Five hub breast cancer target genes associated with 4-NP exposure were screened, containing TP53, HDAC1, ESR1, CTNNB1 and MYC. GO and KEGG analyses revealed that intersecting genes mainly influenced PI3K-Akt signaling, MicroRNAs in cancer, Chemical carcinogenesis−receptor activation and MAPK signaling. Molecular docking confirmed strong binding affinities of 4-NP to these hub genes. Subsequently several high-confidence candidate regulatory miRNAs especially miR-22, -148a, -181a and −152 were identified that shed light on miRNA regulatory mechanisms by which 4-NP increases breast cancer risk.

**Conclusion:**

Our study demonstrates that 4-NP exposure perturbs protein conformational of hub targets, activating cascades and dysregulating signaling pathway to potentiate breast cancer risk. Furthermore, we identify a novel miRNA-mediated regulatory axis alongside MAPK signaling as critical mechanisms underpinning 4-NP toxicity.

## Introduction

As is widely known, 4-Nonylphenol (4-NP) is a class of endocrine-disrupting compounds characterized by their exogenous estrogenic properties and potential carcinogenicity. A primary application of 4-NP lies in the synthesis of plasticizers, surfactants, antioxidants, and emulsifiers. The widespread utilization of surfactants derived from 4-NP contributes significantly to its discharge into wastewater treatment systems or its direct environmental release [1]. Due to their extensive application in both domestic and industrial settings, these compounds are ubiquitously detected in consumer products, including food and drinking water [2]. Despite the implementation of regulatory controls on the levels of nonylphenol (NP) in environmental water sources by certain nations, there persists a significant discharge of NP into the environment [3]. 4-NP is a lipophilic compound that exerts estrogen-like effects by influencing multiple cellular signaling pathways [4]. Previous studies have shown that 4-NP can bind to estrogen receptors, leading to endocrine disruption that subsequently promotes the growth of breast cancer (BRCA) cells [5]. Nevertheless, the potential mechanisms by which it may contribute to BRCA pathogenesis through other pathways, as well as its overall risk assessment for BRCA development, have not been definitively established in the scientific literature.

Breast cancer is the most common female malignancy worldwide [6], the development of which is associated with the interplay of various factors, containing genetics, lifestyle and environmental influences. In recent years, the increasing incidence of BRCA has been suspected to be linked to exposure to multiple chemical pollutants in the environment. Research has shown that the surfactant 4-NP can promote the growth of BRCA cells [5]. However, the exact molecular targets of its action have not yet been fully deciphered. A more comprehensive understanding of the potential molecular mechanisms underlying 4-NP exposure increases BRCA risk could provide new insights for risk prevention.

Network toxicology is a multidisciplinary research paradigm that combines bioinformatics, systems biology and cheminformatics, which provides a holistic framework to elucidate how chemical substances interfere with biological networks and compromise cellular functions, subsequently resulting in disease. In addition, molecular docking technology is a computational method used to predict how molecules interact with each other, particularly focusing on the binding of small molecules (ligands) to larger biomolecules like proteins, enzymes or nucleic acids. It simulates the interaction between these molecules to determine the binding affinity, binding site, and potential mechanism of action [7]. This research aims to employ these cutting-edge technologies to uncover the underlying mechanisms by which 4-NP exposure exerts adverse effects on BRCA. We also seek to provide novel insights into the safety assessment of 4-NP and the prevention of environmental pollutant exposure related to BRCA.

## Methods

### Preliminary prediction of 4-NP toxicity and target identification

The molecular structure and SMILES notation of 4-nonylphenol were retrieved from the PubChem database (https://pubchem.ncbi.nlm.nih.gov/). Preliminary toxicity analysis was conducted using the ProTox (https://tox.charite.de/) and ADMETlab databases (https://admetmesh.scbdd.com/), with all the results documented in the Supplementary Files. Potential molecular targets were identified through comprehensive screening of the Swiss Target Prediction (http://www.swisstargetprediction.ch/), Search Tool for Interactions of Chemicals (STITCH) (http://stitch.embl.de/) and Chemical Database of Bioactive Molecules (ChEMBL) (http://www.ebi.ac.uk/chembl/), with the species parameter specifically set to “Homo sapiens” for target prediction. Name normalization was conducted employing the Uniprot database (https://www.uniprot.org/). Data consolidation was implemented by merging datasets from three sources.

### Acquisition of breast cancer-associated genes

Breast cancer-associated genes were retrieved from the GeneCards database (https://www.genecards.org/), filtered using a relevance score threshold of >10, and subsequently combined with disease targets obtained from the Online Mendelian Inheritance in Man (OMIM) (https://omim.org/) and Therapeutic Target databases (TTD) (http://db.idrblab.net/ttd/) to form a unified gene set. The results from three database were merged and deduplicated separately.

### Construction of the compound-gene regulatory network for BRCA

The intersection of BRCA-associated genes and compound-target interactions was identified and the regulatory network was visualized using Cytoscape software (version 3.10.3).

### Functional and pathway enrichment analyses of intersecting targets

Statistical significance in the Gene Ontology (GO) enrichment analysis on the compound-disease-associated proteins was defined by a Benjamini-Hochberg-adjusted q-value <0.05. KEGG (Kyoto Encyclopedia of Genes and Genomes) is primarily utilized to identify signaling pathways that may be enriched with target genes.

### Construction of Protein-Protein Interaction networks and identification of core genes

The protein-protein interaction (PPI) network was constructed by analyzing target gene interactions using the STRING database (https://string-db.org/) with an interaction score threshold of >0.9. This threshold represents the highest confidence level in STRING, as it minimizes false positives by requiring multiple independent evidence types (e.g., experimental validation, curated databases, and coexpression data), thereby ensuring high biological relevance of the included interactions. We calculated the topological properties of network nodes, such as degree, betweenness centrality and closeness centrality, based on which the genes were ranked and the top 5 genes with the highest degree were selected as the core genes. Visualization was performed using Cytoscape software. (version 3.10.3).

### Validation of differential expression of core genes in BRCA

The differential expression of core genes in BRCA samples from The Cancer Genome Atlas (TCGA) database was validated using the UALCAN platform (https://ualcan.path.uab.edu/), Statistical significance was defined as P-value < 0.01, a stringent threshold selected to minimize false positives and enhance the reliability of identified differentially expressed genes (DEGs) in downstream analyses.

### Molecular docking of 4NP with hub genes

The structure of 4-NP was obtained from the PubChem database. The three-dimensional (3D) structures of target proteins were retrieved from the Protein Data Bank (PDB) database (https://www.rcsb.org/) with the following selection criteria: Organism: Homo sapiens; Experimental method: X-ray diffraction; Resolution≤ 2.0 Å, as structures resolved at this level enable unambiguous determination of atomic positions, including side-chain conformations and ligand-binding geometries. If no structure meeting this criterion was available, the structure with the highest resolution (lowest Å value) for the target protein was selected. Based on the established criteria, suitable 3D structures for the following five proteins were selected: Tumor Protein P53 (TP53; PDB ID: 1KZY); Histone Deacetylase 1 (HDAC1; PDB ID: 5ICN); Estrogen Receptor 1 (ESR1; PDB ID: 1SJ0); Catenin Beta-1 (CTNNB1; PDB ID: 1LUJ) and MYC Proto-Oncogene Protein (MYC; PDB ID: 1NKP). Following protein preparation in PyMOL2.4 involved removal of water molecules. The binding site of the target protein was identified through computational prediction using tools like PrankWeb to locate conserved residues and energetic hotspots [8]. Molecular docking was performed using AutoDock Vina, which evaluates binding affinity based on empirical energy terms (including gaussian steric repulsion, hydrogen bonding, and hydrophobic interactions). Ligand conformations were ranked by predicted binding energy (ΔG in kcal/mol), with poses exhibiting ΔG ≤ −6.0 kcal/mol and clustering frequency≥40% selected as high-affinity binding modes [8]. Resultant complexes were visualized in PyMOL2.4, while Discovery Studio 2019 software generated detailed 2D interaction diagrams highlighting key residue contributions.

### Prediction of miRNA targeting the regulation of core genes

Given the significant involvement of the core genes in the “MicroRNAs in cancer” pathway as indicated by KEGG analysis, we utilized the miRDB database (https://mirdb.org) which employs a machine learning algorithm to predict miRNAs targeting these core genes. Stringent thresholds were applied to enhance prediction reliability: (1) Target Score ≥85 (excluding predictions below this threshold to prioritize high-confidence interactions with optimal seed matching and binding site accessibility); (2) exclusion of miRNAs with >2000 predicted targets genome-wide (mitigating non-specific regulators lacking functional focus, as human miRNAs average ~606 targets); and (3) restriction to human species [9]. The identified target miRNAs were subjected to WIKIPathways enrichment analysis and visualized using the Custom Heatmap Calculator tool available on the miRPathDB platform. Representative miRNAs were selected based on two criteria: (1) Significant enrichment (FDR < 0.05) in ≥3 KEGG/WIKIPathways; (2) Targeting core genes functionally linked to 4-NP exposure phenotypes (e.g., oncogenesis, DNA repair), and their target genes were identified from prior miRNA target prediction analyses. The differential expression of these miRNAs in breast cancer was further validated using the dbDEMC 3.0 database (employing limma for microarray data and edgeR/DESeq2 for sequencing data, with Benjamini-Hochberg-adjusted P values < 0.05 and |log2FC| ≥ 1 as significance criteria) [10].

## Results

### Structure acquisition and toxicity prediction of 4-NP

**Fig 1:**
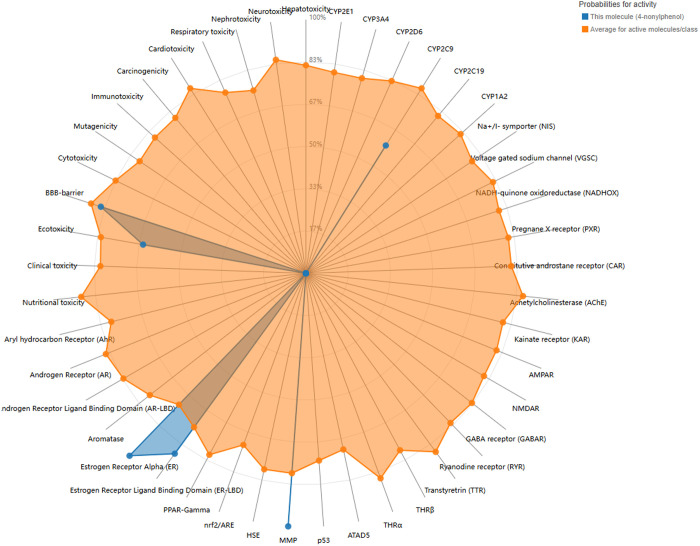
Toxicity prediction of 4-Nonylphenol.

The 3D structure and SMILE notation (CCCCCCCCCC1 = CC = C(C = C1)O) of 4-NP were obtained from the PubChem database. Toxicity prediction revealed that 4-NP exhibits significant activity across several clusters, including estrogen receptor (ER), estrogen receptor ligand binging domain (ER-LBD) and matrix metalloproteinase (MMP), all of which may be implicated in the pathogenesis of breast cancer (Fig 1) and all ProTox -predicted toxicological data for 4-NP have been deposited in Supporting Information S1 Table.

### Acquisition of target genes of 4-NP

**Fig 2:**
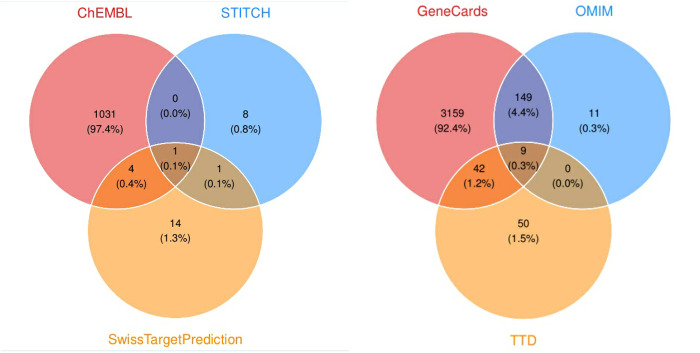
Collection of 4-NP and breast cancer targets.

Through the utilization of the Swiss Target Prediction, STITCH and ChEMBL databases, a total of 14, 8 and 1031 target genes of 4-NP were identified, respectively. The union of these targets was subsequently computed (Fig 2).

### Compound-BRCA-associated target genes and regulatory network

**Fig 3:**
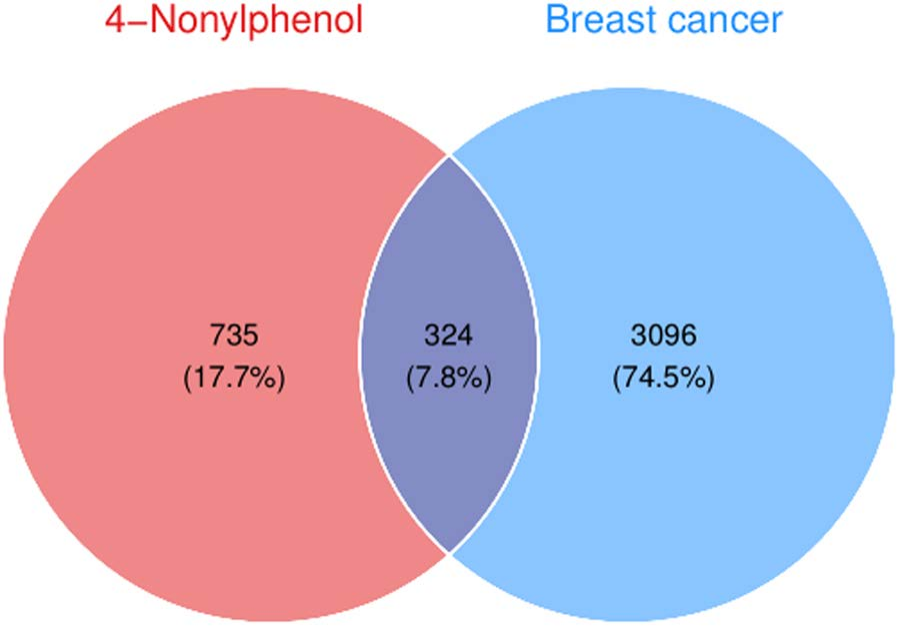
Intersection targets of 4-Nonylphenol and breast cancer.

**Fig 4:**
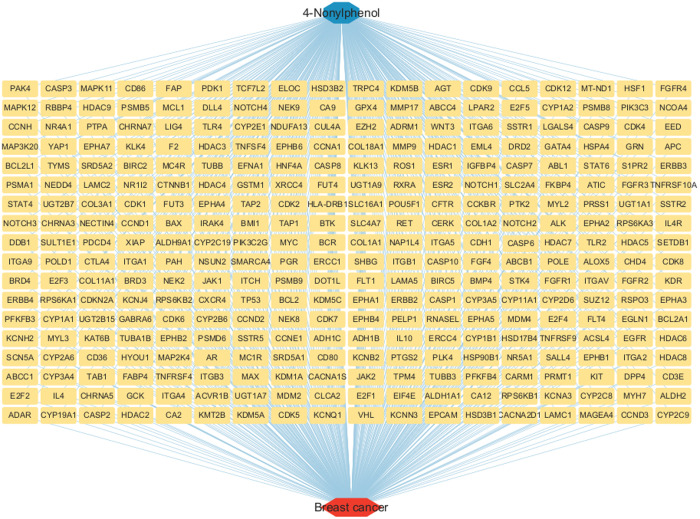
Regulatory network between 4-NP and breast cancer.

A comprehensive compilation of 3420 BRCA-associated genes was procured from the GeneCards, OMIM and TTD databases, as illustrated in Fig 2. The intersection of these genes with the targets of 4-NP yielded a total of 324 proteins (Fig 3), by which a regulatory network was constructed as shown in Fig 4.

### Enrichment analysis of intersecting target genes

**Fig 5:**
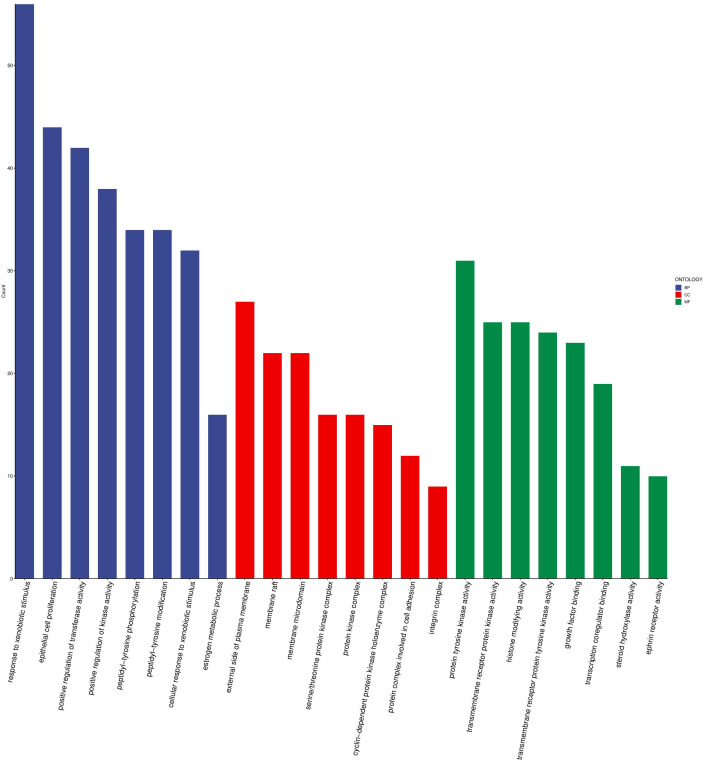
Gene Ontology (GO) term analysis plot Histogram shows the top 8 enriched entries for each GO category (BP, CC and MF). The height of each bar corresponds to the gene count, reflecting the degree of enrichment within the respective category. These entries stress critical biological processes and cells composition and molecular function that may be affected by 4-NP exposure.

**Fig 6:**
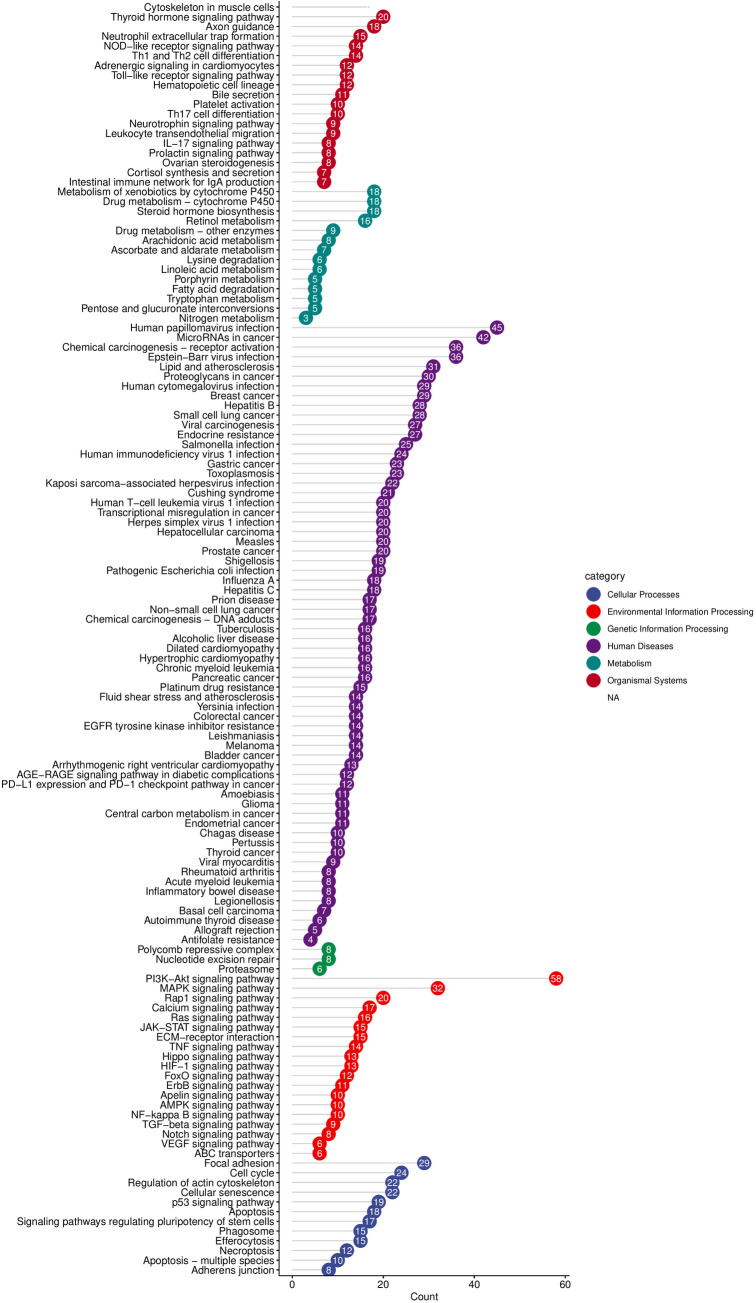
Kyoto Encyclopedia of Genes and Genomes (KEGG) analysis identifies signaling pathways that may be enriched with target genes. Each bubble corresponds to the gene expression in a specific pathway. The color of the bubble corresponds to a certain category. The size of the number in the bubble corresponds to the gene count, reflecting the degree of enrichment within the respective category.

The 324 overlapping genes identified were considered as potential targets through which 4-NP may initiate BRCA. GO analysis demonstrated that these target genes were significantly enriched in several biological processes, including response to xenobiotic stimulus, epithelial cell proliferation and positive regulation of transferase activity (Fig 5). At the molecular function level, these genes were predominantly associated with protein tyrosine kinase activity, indicating their potential involvement in signal transduction pathways. KEGG pathway analysis revealed that the compound was most likely to initiate BRCA through several key pathways, including the phosphatidylinositol 3-kinase-protein kinase B (PI3K-Akt) signaling pathway, MicroRNAs in cancer and Chemical carcinogenesis − receptor activation (Fig 6).

### Identification of core genes

**Fig 7:**
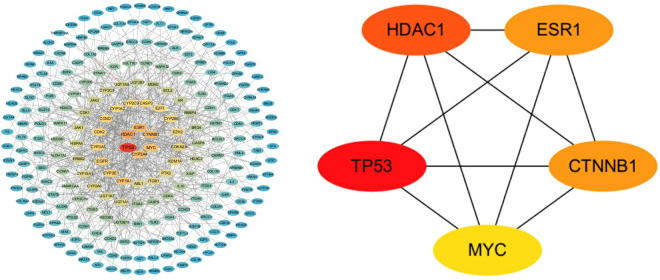
The PPI networks and the core genes.

The PPI network of potential targets was constructed using Cytoscape software (version 3.10.3). Based on network topology analysis, six genes TP53, HDAC1, ESR1, CTNNB1, MYC and CYP3A4 with the highest degree scores were initially identified [11]. However, CYP3A4 was subsequently excluded from the analysis due to its lack of direct interactions with the other five genes. The remaining genes were considered as potential core genes, which were visualized in Fig 7, with the details documented in the Supporting Information (S2 Table), demonstrating their central positions and extensive connectivity within the network.

### Validation of differential expression of core genes in breast cancer

**Fig 8:**
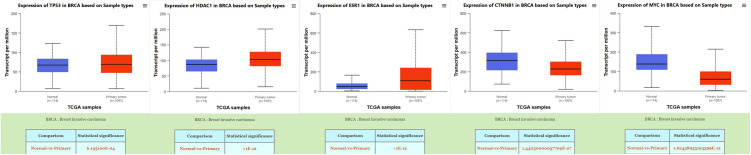
Differential expression of core genes in breast cancer samples from TCGA database.

The differential expression of the core genes was validated in breast cancer samples from TCGA database using the UALCAN platform. Analysis revealed that all core genes exhibited statistically significant differential expression profiles with p-values < 0.01, as illustrated in Fig 8.

### Molecular docking

Stable binding of 4-NP to five target proteins (TP53, HDAC1, ESR1, CTNNB1, and MYC) was demonstrated by molecular docking, with calculated binding affinity of −4.8, −6.8, −5.1, −5.1, and −5.9 kcal/mol, respectively. Binding affinity, quantitatively expressed as the negative logarithm of the dissociation constant, serves as a crucial indicator of molecular interaction strength between ligands and their corresponding receptors. The magnitude of this negative value directly correlates with the binding strength, wherein a larger absolute value denotes more robust molecular interactions and higher binding stability. Notably, four out of the five interactions demonstrated binding affinity below the threshold of −5.0 kcal/mol, indicating favorable and stable binding conformations. The strongest interaction (HDAC1) was observed with a binding affinity of −6.8 kcal/mol, suggesting a particularly robust molecular association.

**Fig 9:**
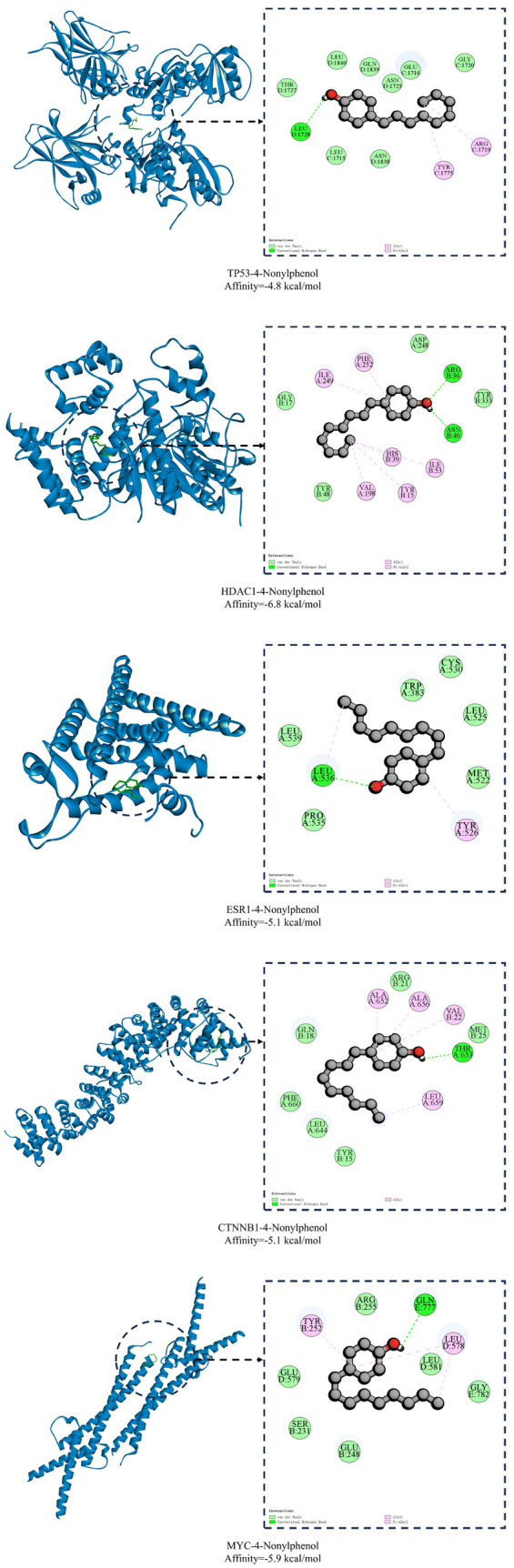
Molecular docking of 4-NP and core genes.

In the 2D interaction diagrams, the circles represent the amino acid residues involved in the interactions between the proteins and 4-NP. The dashed lines denote the interaction forces. Hydrogen bonding play significant roles in facilitating the binding of 4-NP to its target protein. As illustrated in Fig 9, 4-NP forms hydrogen bonds with specific residues across five target proteins: TP53: Leu1728 in Chain D, HDAC1: Arg36 and Asn40 in Chain B, ESR1: Leu536 in Chain A, CTNNB1: Thr653 in Chain A, MYC: Gln777 in Chain E. Concurrently, pink dashed lines in Fig 9 represent non-covalent interactions, primarily Alkyl (attraction between ligand alkyl groups and hydrophobic protein pockets, driven by entropic effects and van der Waals forces) and Pi-Alkyl (electrostatic and hydrophobic interactions between aromatic π-systems and aliphatic side chains of residues like Val/Leu/Ile). These forces significantly enhance binding stability by excluding solvent from hydrophobic interfaces and improving shape complementarity. Though individually modest, they collectively reinforce ligand specificity and complex longevity, particularly when synergizing with hydrogen bonds in active sites. The Root-mean-square deviation (RMSD) values characterizing ligand binding to each protein are provided in S3 Table (Supporting Information).

### MicroRNAs targeting the hub genes

**Fig 10:**
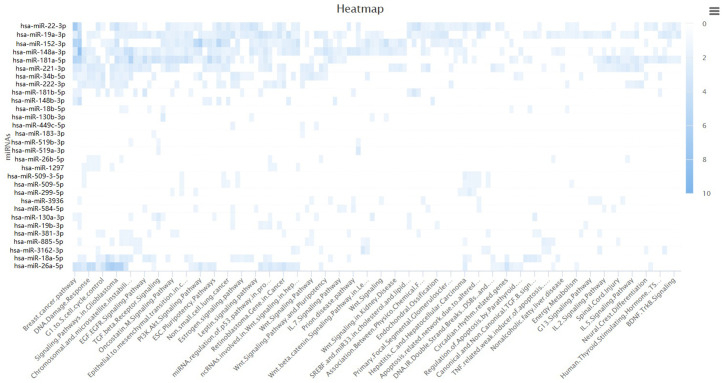
WIKIPathways of miRNA targeting core genes.

**Fig 11:**
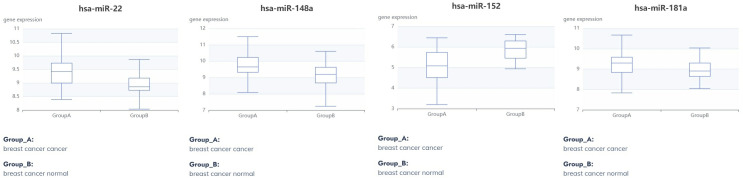
Differential expression of miRNAs targeting core genes in breast cancer.

Using the miRDB online tool, we predicted 45 miRNAs that potentially regulate the core genes. Full prediction details (including miRDB scores and target genes) are provided in Supporting Information S4 Table. Subsequent WIKIPathways enrichment analysis revealed that several miRNAs were significantly enriched in pathways associated with breast cancer (hsa-miR-22-3p, hsa-miR-181a-5p and hsa-miR-152-3p), DNA damage response (hsa-miR-152-3p and has-miR-148a-3p) and PI3K-Akt signaling (hsa-miR-152-3p), indicating their roles as upstream/downstream effectors in 4-NP-induced breast cancer. For Fig 10 visualization, miRNAs significantly enriched in ≥3 pathways were prioritized. This criterion was adopted because multi-pathway enrichment suggests a “core regulatory hub” property—such miRNAs are more likely to coordinate multiple biological processes (e.g., proliferation, DNA repair, and signal transduction), thereby providing a holistic mechanistic explanation for 4-NP’s effects. However, we acknowledge that other miRNAs with high prediction scores (e.g., miRDB score >95) or literature-supported regulatory evidence may exist even if enriched in fewer pathways. The target genes of these miRNAs were exclusively identified as ESR1, as presented in Table 1. These miRNAs exhibited significant differential expression (Adjust P-value<0.05) between breast cancer and normal tissues, with the results visualized in Fig 11 and detailed in Table 2.

**Table 1:** The targeted gene of miRNA.

Target Score	miRNA Name	Gene Symbol
97	hsa-miR-22-3p	ESR1
96	hsa-miR-148a-3p	ESR1
96	hsa-miR-152-3p	ESR1
86	hsa-miR-181a-5p	ESR1

**Table 2:** Differential expression of targeting miRNAs between breast cancer and normal tissues.

miRNA ID	Design	Log2FC	AveExpr	adj Pvalue	Status
hsa-miR-22	cancer vs normal	0.42	9.19	5.00E-02	UP
hsa-miR-148a	cancer vs normal	0.81	9.34	8.00E-05	UP
hsa-miR-152	cancer vs normal	−0.8	5.18	1.46E-02	DOWN
hsa-miR-181a	cancer vs normal	0.33	9.12	5.59E-03	UP

The target genes of these miRNAs were exclusively identified as ESR1.

These miRNAs exhibited significant differential expression (Adjust P-value<0.05) between breast cancer and normal tissues.

## Discussion

Breast cancer incidence in developing nations exhibits a progressive upward trajectory in recent epidemiological records [12,13]. Intriguingly, urban populations demonstrate higher prevalence rates compared to rural demographics [14]. Furthermore, epidemiological migration studies demonstrate that female migrants from low-incidence regions acquire elevated cancer risk upon prolonged residence in high-incidence zones, strongly implicating environmental determinants as critical modulators in BRCA [15].

4-Nonylphenol (4-NP), a representative alkylphenol compound, which is extensively used in industrial applications, such as plasticizer, surfactant and emulsifier, also serves as a precursor in fragrance synthesis and antioxidant manufacturing, resulting in widespread human and environmental exposure [16]. As a prototypical endocrine-disrupting chemical (EDC), 4-NP exhibit estrogenic properties through xenohormone interactions, though their carcinogenic classification remains controversial. Notably, 4-NP exposure has been documented to promote cervical carcinogenesis through MAPK signaling pathway [17]. Emerging evidence from in vitro models reveals its role as an estrogen receptor alpha (ERα) agonist in MCF-7 human BRCA cells, suggesting potential involvement in BRCA progression [18]. However, the comprehensive molecular pathophysiology underlying 4-NP-associated breast oncogenesis remains largely unexplored.

Our study aims to provide novel insights into the molecular mechanisms by which 4-NP contributes to BRCA. Based on preliminary findings utilizing network toxicology, we have identified that 4-NP may mediate its potential carcinogenic effects in breast cancer through dysregulation of estrogen receptor signaling and disruption of MMP activity. Furthermore, five hub genes (TP53, HDAC1, ESR1, CTNNB1 and MYC) played critical roles in mediating 4-NP-induced BRCA.

TP53, a pivotal tumor suppressor protein, plays a central role in regulating DNA damage repair and DNA replication stress, thereby safeguarding genomic stability [19,20]. TP53 mutations critically drive tumorigenesis through loss-of-function (LOF) in tumor suppression and/or acquisition of gain-of-function (GOF) oncogenic activity [21,22]. We hypothesize that 4-NP binding to TP53 induces conformational alterations that compromise its DNA damage surveillance capacity. Concurrently, mutant TP53 may acquire novel oncogenic properties, such as activation of the PI3K-Akt signaling pathway drives metabolic reprogramming by upregulating aerobic glycolysis (the Warburg effect), thereby supplying cancer cells with ATP and biosynthetic precursors to fuel their uncontrolled proliferation-a process further modulated by miRNA networks [23]. Enrichment analysis (Fig 5B) demonstrated significant enrichment of 4-NP-associated target genes in ‘MicroRNAs in cancer’ relevant to breast cancer. Complementary WikiPathways analysis (Fig 9) revealed heightened activity of core target miRNAs—notably hsa-miR-152-3p—in breast cancer-associated processes, DNA damage response, and PI3K-Akt signaling. Collectively, these findings suggest 4-NP may promote breast carcinogenesis through a sequential pathway involving: TP53 dysregulation → miRNA aberrations (e.g., hsa-miR-152-3p) → PI3K-Akt signaling hyperactivation.

Emerging evidence indicates that HDAC1 dysregulation induces aberrant cell cycle progression [24] and plays a pivotal regulatory role in multiple biological processes, including DNA damage response and cellular proliferation/differentiation processes [25–27]. The molecular pathogenesis appears to involve HDAC1’s direct interaction with the TP53, which subsequently elevates matrix metalloproteinase (MMP)-2 and MMP-9 enzymatic activity [28,29]. This observation aligns with our toxicity predictions indicating 4-Nitrophenol (4-NP) activity at MMP targets. We propose a mechanistic axis wherein 4-NP binds to HDAC1, subsequently modulating TP53 to activate MMPs. This proteolytic cascade promotes recruitment of pro-tumorigenic immune cells—specifically M2-polarized macrophages—establishing an immunosuppressive microenvironment conducive to carcinogenesis. Collectively, these interactions delineate a 4-NP→HDAC1 → TP53 → MMP→Immunosuppression→Oncogenesis signaling axis.

Molecular docking analyses indicate that 4-NP forms stable interactions with HDAC1 through two hydrogen bonds. This binding may induce conformational changes in HDAC1’s core structure, potentially inhibiting its enzymatic activity and reducing histone deacetylation. Subsequent hypoacetylation is predicted to trigger the transcriptional derepression of the proto-oncogene MYC, thereby promoting breast tumorigenesis—a mechanism corroborated by the observed regulatory interplay among HDAC1, MYC, and TP53 in Fig 6B. MYC acts as a master regulator of cellular proliferation, apoptosis, and tumor progression via interactions with transcription factors and downstream targets [30,31]. Furthermore, 4-NP exhibits favorable binding with MYC itself, suggesting dual oncogenic roles: (1) Indirect amplification through the HDAC1 → hypoacetylation→MYC activation cascade, positioning MYC as a downstream effector; (2) Direct binding-driven MYC dysregulation, functioning as an autonomous signaling amplifier in 4-NP-induced breast carcinogenesis.

4-NP exhibits estrogenic activity capable of binding to estrogen receptors (ERs) and potentially triggering diverse pathological outcomes. Specifically, experimental studies confirm that 4-NP acts as an agonist of estrogen receptor α (ERα) in MCF-7 human breast cancer cells, thereby promoting estrogenic signaling pathways implicated in breast carcinogenesis [18]. However, our study reveals a novel dimension to this mechanism. Through miRDB prediction and WIKIPathway enrichment analysis, four ESR1-targeting miRNAs were enriched in breast cancer pathway (hsa-miR-22, -181a, −152), DNA damage response (has-miR-152, 148a) and PI3K-Akt signaling pathway (has-miR-152), with hsa-miR-22, -181a and -148a upregulated and hsa-miR-152 downregulated in breast cancer. Notably, miR-181a has been implicated in suppressing DNA damage repair and modulating autophagy to sustain cell survival across various cancers [32,33]. 4-NP binds to ESR1, potentially inducing conformational changes that activate aberrant signaling. This dysregulation upregulates oncogenic miRNAs (miR-22, miR-181a, miR-148a), which collectively promote DNA damage while downregulating miR-152. The suppression of miR-152 relieves its inhibitory effect on PIK3CA, thereby activating the PI3K-Akt pathway. These synergistic perturbations elevate the risk of breast carcinogenesis. This discovery provides new insights into the complex interplay between protein-ligand interactions, potentially expanding our understanding of chemical-induced carcinogenesis.

CTNNB1, the gene encoding β-catenin, mutations in which can lead to conformational changes of the β-catenin protein, resulting in aberrant activation of the Wnt-β-catenin signaling pathway [34]. This activation subsequently upregulates the expression of the pro-proliferative gene c-MYC, sequentially modulating cell proliferation and contributing to tumorigenesis [35]. Additionally, emerging evidence suggests that this signaling pathway is associated with immune suppression within the tumor microenvironment [36]. As illustrated in Fig 6B, CTNNB1 and three additional core genes form a signaling cascade with MYC, amplifying pro-proliferative signaling in breast cancer cells. In this cascade, MYC functions as both a transcriptional amplifier and effector molecule, driving the expression of downstream targets (e.g., CCND1, CDK4/6) that accelerate G1/S phase transition [37]. Critically, this CTNNB1/TP53/ESR1/HDAC1-MYC axis operates as a primary mechanism for 4-NP-induced breast carcinogenesis, distinct from other endocrine-disrupting chemicals (EDCs) that predominantly activate the monotonic estrogen receptor α (ERα) pathway.

Notably, KEGG enrichment analysis (Fig 5B) revealed significant enrichment of intersection genes within the MAPK signaling pathway. Prior evidence demonstrated that 4-NP exposure promoted cervical carcinogenesis through MAPK hyperactivation [17,38]. Collectively, these findings suggest that MAPK pathway dysregulation may contribute to 4-NP-induced breast cancer pathogenesis. Critically, this multi-pathway activation paradigm—involving both estrogenic signaling and MAPK cascades—distinguishes 4-NP from other EDCs that primarily rely on singular ERα-mediated pathways. Consequently, such mechanistic complexity may potentiate higher cumulative breast cancer risk under chronic 4-NP exposure scenarios.

As illustrated in Fig 7, our analysis revealed significant differential expression patterns of these five core genes between BRCA and normal tissues. Notably, the expression alterations were not uniformly uprted, demonstrating a heterogeneous regulatory pattern. This observation further supports our hypothesis that 4-NP predominantly exerts its effects through inducing genetic mutations that subsequently lead to protein conformational changes, rather than merely stimulating gene amplification. The diverse expression profiles observed across these core genes provide compelling evidence for this mutation-driven mechanism of action. Therefore, elucidating the molecular interaction patterns between proteins and 4-NP is critical for deciphering the compound’s pathogenic mechanisms at the molecular level.

However, the dose-dependent responses of these genes to 4-NP exposure remain poorly characterized, necessitating further interdisciplinary investigations encompassing epidemiological studies, clinical research and molecular biology approaches. Such comprehensive studies could provide valuable insights for developing more effective preventive strategies.

Furthermore, our investigation extended to miRNA networks regulated by ESR1. While the potential for environmental toxicants to directly target miRNA-mediated regulatory processes in disease pathogenesis remains to be fully elucidated, this emerging area of research offers novel perspectives for understanding the mechanisms underlying environment-induced diseases. The exploration of miRNA-mediated pathways may reveal additional molecular targets and provide new avenues for investigating environmental exposure-related disease mechanisms.

## Conclusion

In summary, our study demonstrates that 4-nonylphenol (4-NP) perturbs breast carcinogenesis through multi-target interactions, signaling cascades, and pathway dysregulation. Beyond directly amplifying the proto-oncogene MYC, 4-NP binds to TP53, HDAC1, CTNNB1, and ESR1, forming a coordinated oncogenic cascade that amplifies MYC-driven proliferative signals. This multi-faceted perturbation likely involves the activation of PI3K-Akt signaling and disruption of pathways governing DNA damage repair and cell cycle progression. Notably, the implicated MAPK pathway activation distinguishes 4-NP from other EDCs, which predominantly rely on monotonic ERα signaling. This mechanistic complexity warrants further exploration to delineate 4-NP-specific oncogenic networks.

These molecular interactions underscore the importance of reducing environmental exposure to surfactants like 4-NP as a potential strategy for breast cancer risk mitigation. Our studies strongly advocate for the adoption of safer alternatives to minimize environmental and human exposure. Furthermore, this study aims to contribute to the development of evidence-based interventions and policies aimed at reducing BRCA risk.

## Supporting information

S1 Table ProTox-predicted toxicological data for 4-NP. This table presents ProTox-derived predictions of 4-NP toxicity, including activity status (active/inactive) and prediction probability across all targets.
(XLSX)

S2 Table Potential protein interactions of core genes. This table summarizes the functional metrics of six computationally identified hub genes within the protein-protein interaction (PPI) network, derived from topological analysis of network properties.
(XLS)

S3 Table RMSD values of ligand binding to protein. Based on the computational analysis of protein-ligand docking results, the table presents the binding affinity of five proteins complexed with ligands across nine distinct conformational states (modes). Two structural deviation metrics are reported for each mode: RMSD l.b., and RMSD u.b., which quantify conformational variations relative to the optimal binding pose.
(XLSX)

S4 Table Prediction of miRNAs targeting core proteins. This table presents computationally predicted miRNAs that target core regulatory genes, along with their corresponding protein and prediction confidence scores derived from the miRDB database.
(XLSX)
